# Mutation p.R356Q in the Collybistin Phosphoinositide Binding Site Is Associated With Mild Intellectual Disability

**DOI:** 10.3389/fnmol.2019.00060

**Published:** 2019-03-12

**Authors:** Tzu-Ting Chiou, Philip Long, Alexandra Schumann-Gillett, Venkateswarlu Kanamarlapudi, Stefan A. Haas, Kirsten Harvey, Megan L. O’Mara, Angel L. De Blas, Vera M. Kalscheuer, Robert J. Harvey

**Affiliations:** ^1^Department of Physiology and Neurobiology, University of Connecticut, Storrs, CT, United States; ^2^Department of Pharmacology, UCL School of Pharmacy, London, United Kingdom; ^3^Research School of Chemistry, The Australian National University, Canberra, ACT, Australia; ^4^Institute of Life Science, School of Medicine, Swansea University, Singleton Park, Swansea, United Kingdom; ^5^Department of Computational Molecular Biology, Max Planck Institute for Molecular Genetics, Berlin, Germany; ^6^Group Development and Disease, Max Planck Institute for Molecular Genetics, Berlin, Germany; ^7^School of Health and Sport Sciences, University of the Sunshine Coast, Sippy Downs, QLD, Australia; ^8^Sunshine Coast Health Institute, Birtinya, QLD, Australia

**Keywords:** *ARHGEF9*, Collybistin, Gephyrin, PH domain, PI3P, XLID

## Abstract

The recruitment of inhibitory GABA_A_ receptors to neuronal synapses requires a complex interplay between receptors, neuroligins, the scaffolding protein gephyrin and the GDP-GTP exchange factor collybistin (CB). Collybistin is regulated by protein-protein interactions at the N-terminal SH3 domain, which can bind neuroligins 2/4 and the GABA_A_R α2 subunit. Collybistin also harbors a RhoGEF domain which mediates interactions with gephyrin and catalyzes GDP-GTP exchange on Cdc42. Lastly, collybistin has a pleckstrin homology (PH) domain, which binds phosphoinositides, such as phosphatidylinositol 3-phosphate (PI3P/PtdIns3P) and phosphatidylinositol 4-monophosphate (PI4P/PtdIns4P). PI3P located in early/sorting endosomes has recently been shown to regulate the postsynaptic clustering of gephyrin and GABA_A_ receptors and consequently the strength of inhibitory synapses in cultured hippocampal neurons. This process is disrupted by mutations in the collybistin gene (*ARHGEF9*), which cause X-linked intellectual disability (XLID) by a variety of mechanisms converging on disrupted gephyrin and GABA_A_ receptor clustering at central synapses. Here we report a novel missense mutation (chrX:62875607C>T, p.R356Q) in *ARHGEF9* that affects one of the two paired arginine residues in the PH domain that were predicted to be vital for binding phosphoinositides. Functional assays revealed that recombinant collybistin CB3_SH3-_^R356Q^ was deficient in PI3P binding and was not able to translocate EGFP-gephyrin to submembrane microaggregates in an *in vitro* clustering assay. Expression of the PI3P-binding mutants CB3_SH3-_^R356Q^ and CB3_SH3-_^R356N/R357N^ in cultured hippocampal neurones revealed that the mutant proteins did not accumulate at inhibitory synapses, but instead resulted in a clear decrease in the overall number of synaptic gephyrin clusters compared to controls. Molecular dynamics simulations suggest that the p.R356Q substitution influences PI3P binding by altering the range of structural conformations adopted by collybistin. Taken together, these results suggest that the p.R356Q mutation in *ARHGEF9* is the underlying cause of XLID in the probands, disrupting gephyrin clustering at inhibitory GABAergic synapses *via* loss of collybistin PH domain phosphoinositide binding.

## Introduction

The Dbl-family guanine nucleotide exchange factor collybistin was initially identified as an interactor of the inhibitory postsynaptic clustering protein gephyrin (Kins et al., 2000). Collybistin exists in several splice isoforms differing in the N- or C-termini (CB1-CB3), and the presence or absence of an exon encoding a regulatory SH3 domain (e.g., CB3_SH3+_, CB2_SH3-_; Kins et al., 2000; Harvey et al., 2004). Collybistin variants lacking the SH3 domain (e.g., CB2_SH3-_/CB3_SH3-_) are capable of forming submembrane gephyrin microclusters in cellular models (Kins et al., 2000; Harvey et al., 2004). By contrast, collybistin variants containing SH3 domain (e.g., CB2_SH3+_/CB3_SH3+_) do not trigger clustering, but co-localize with gephyrin in large intracellular aggregates (Kins et al., 2000; Harvey et al., 2004). Later studies revealed that SH3 domain containing variants adopt a closed and autoinhibited conformation that largely prevents membrane binding (Soykan et al., 2014). Since collybistin variants containing SH3 domains predominate in the brain and spinal cord (Harvey et al., 2004; Soykan et al., 2014) this triggered a search for neuronal regulatory proteins that could bind to the SH3 domain and trigger collybistin activity. This resulted in the identification of neuroligin-2 (NL2), neuroligin-4 (NL4) and the GABA_A_ receptor α2 subunit as collybistin SH3 domain interactors (Poulopoulos et al., 2009; Saiepour et al., 2010; Hoon et al., 2011). The intracellular domains of these proteins contain collybistin-binding motifs that interact with the SH3 domain, triggering collybistin-mediated gephyrin clustering by conformational “opening” of SH3 domain containing collybistin variants. Consistent with these findings, knockout mice lacking collybistin, or collybistin activators, show a brain region specific loss of gephyrin clusters at inhibitory synapses and defects in inhibitory synaptic transmission (Papadopoulos et al., 2007, 2008; Jedlicka et al., 2009, 2011; Poulopoulos et al., 2009; Hoon et al., 2011; Panzanelli et al., 2011). Disruption of collybistin-GABA_A_ receptor α2 subunit interactions in mice also leads to loss of a subset of inhibitory synapses, spontaneous seizures and early mortality, with surviving animals showing anxiety-like behavior (Hines et al., 2018).

All known collybistin isoforms also contain tandem RhoGEF and pleckstrin-homology (PH) domains. The RhoGEF domain catalyzes GDP-GTP exchange on the small GTPase Cdc42 and also mediates interactions with gephyrin (Xiang et al., 2006). However, the role of RhoGEF activity on Cdc42 in gephyrin clustering is unclear. Gephyrin binding inhibits collybistin activity on Cdc42 (Xiang et al., 2006) implying that gephyrin binding inhibits collybistin GDP-GTP exchange. Moreover, artificial collybistin mutants lacking RhoGEF activity on Cdc42 (T91A, K192A and N232A-N233A) are still capable of inducing the formation of submembrane gephyrin clusters in transfected cells and in cultured hippocampal neurons (Reddy-Alla et al., 2010). Lastly, gephyrin and GABA_A_R clustering is not affected by hippocampal deletion of Cdc42 in mice (Reddy-Alla et al., 2010). By contrast, the PH domain plays a more vital role in clustering, since deletion of the collybistin PH domain, or mutation of two key arginine residues (R356/R357) abolishes collybistin-mediated gephyrin clustering in functional assays (Harvey et al., 2004; Kalscheuer et al., 2009; Reddy-Alla et al., 2010). This is because the PH domain and in particular R356/R357 are key determinants of binding of phosphoinositides, including phosphatidylinositol 3-phosphate (PI3P/PtdIns3P; Kalscheuer et al., 2009; Reddy-Alla et al., 2010) and phosphatidylinositol 4-monophosphate (PI4P/PtdIns4P; Ludolphs et al., 2016). Consistent with this view, PI3P located in early/sorting endosomes has recently been shown to regulate the postsynaptic clustering of gephyrin and GABA_A_ receptors and consequently the strength of inhibitory synapses in cultured hippocampal neurons (Papadopoulos et al., 2017).

The critical role of collybistin in inhibitory synaptic structure and function was confirmed by the discovery of missense and nonsense mutations, deletions and complex re-arrangements affecting the collybistin gene (*ARHGEF9*) in patients with X-linked intellectual disability (XLID; Harvey et al., 2004; Marco et al., 2008; Kalscheuer et al., 2009; Lesca et al., 2011; Shimojima et al., 2011; Lemke et al., 2012; de Ligt et al., 2012; Long et al., 2016; Alber et al., 2017; Klein et al., 2017; Wang et al., 2018). However, the associated clinical phenotypes vary significantly, perhaps depending on whether mutant proteins can act in a dominant-negative manner, or other factors, such as skewed X-inactivation in females or the involvement of other nearby genes in chromosomal deletions or rearrangements (Long et al., 2016; Alber et al., 2017; Aarabi et al., 2018). For example, a p.G55A mutation in the SH3 domain acted in a dominant-negative manner, disrupting gephyrin clustering in transfected cells and neurons by affecting GABA_A_R α2 subunit and NL2/NL4 interactions (Harvey et al., 2004; Poulopoulos et al., 2009; Saiepour et al., 2010; Hoon et al., 2011). This mutation was associated with hyperekplexia, early infantile epileptic encephalopathy and severe psychomotor retardation (Harvey et al., 2004). By contrast, a balanced chromosomal translocation resulting in collybistin isoforms that lacked a complete PH domain resulted in disrupted synaptic localization of endogenous gephyrin and GABA_A_ receptors (Kalscheuer et al., 2009). This mutation was associated with a disturbed sleep-wake cycle, increased anxiety and aggressive behavior. However, more recently a series of missense mutations in *ARHGEF9* have been shown to impact collybistin phosphoinositide binding (Papadopoulos et al., 2015; Long et al., 2016). These include p.R290H and p.R338W missense mutations in the RhoGEF domain, which were linked to XLID/epilepsy and non-syndromic (NS)-XLID with variable macrocephaly and macro-orchidism, respectively. Substitution p.R290H was predicted to alter the strength of intramolecular interactions between the RhoGEF and PH domains, while p.R338W was predicted to result in clashes with adjacent amino acids (K363 and N335) and disruption of electrostatic potential and local folding of the PH domain. Thus, both mutations *indirectly* result in a loss of PI3P binding affinity and collybistin-mediated gephyrin clustering (Papadopoulos et al., 2015; Long et al., 2016). In this study, we report the identification of a novel pathogenic missense variant in *ARHGEF9* using next-generation sequencing and variant filtering in a family with mild NS-XLID, which was recently included in a case series (Alber et al., 2017). The identified mutation (p.R356Q) *directly* affects one of the two paired arginine residues in the PH domain that are vital for binding phosphoinositides. Using a combination of PI3P binding assays, gephyrin clustering assays, and molecular dynamics simulations, we present compelling evidence that this mutation not only disrupts phosphoinositide binding, but also results in defective gephyrin clustering in both cellular and neuronal models.

## Materials and Methods

### Exon Capture and DNA Sequencing

X-chromosome exome resequencing and bioinformatics analysis was performed as recently described (Hu et al., 2014, 2016). However, for mapping of the 101bp reads BWA (version 0.5.9-r16, maximal mismatches: -*n* 5) was applied, partial mapping was still performed by using SplazerS (Emde et al., 2012). Genomic DNA from the affected male II:8 was used for constructing the sequencing library using the Illumina Genomic DNA Single End Sample Prep kit (Illumina, San Diego, CA, USA). Enrichment of the X-chromosome exome was then performed using the Agilent SureSelect Human X Chromosome Kit (Agilent, Santa Clara, CA, USA). PCR primers for mutation confirmation and segregation analysis were *ARHGEF9*-D228F 5’-TTTTTCCTCCAGCTTCTTGG-3’ and *ARHGEF9*-D228R 5’-AACCAACCCCCATTGGTACT-3’. This study was carried out in accordance with the recommendations of the University of Welfare and Rehabilitation Sciences in Iran with written informed consent from all subjects. All subjects gave written informed consent in accordance with the Declaration of Helsinki. The protocol was approved by the University of Welfare and Rehabilitation Sciences in Iran.

### Site-Directed Mutagenesis and Expression Constructs

Full-length human collybistin (CB) cDNAs were cloned into the vector pRK5 as previously described (Kalscheuer et al., 2009). Mutations were introduced into pRK5myc-CB3_SH3-_ construct using the QuikChange site-directed mutagenesis kit (Agilent, Santa Clara, CA, USA) and confirmed by Sanger DNA sequencing of the entire coding region.

### PI3P Pull-Down Assays

Affinity purification assays using PI3P agarose beads were carried out as described previously (Kanamarlapudi, 2014). Human embryonic kidney 293 (HEK293) cells were grown in DMEM supplemented with 10% (v/v) fetal bovine serum at 37°C, 5% CO_2_ and transfected with 4 μg pRK5myc-CB3_SH3-_ (wild-type), pRK5myc-CB3_SH3-_^R356Q^, pRK5myc-CB3_SH3-_^R290H^ (XLID mutants) or pRK5myc-CB3_SH3-_^R356N/R357N^ (artificial phosphoinositide binding site mutant, Reddy-Alla et al., 2010) using JetPrime transection reagent (Polyplus; 2 μl/μg DNA). After 48 h, transfected cells were solubilized in a buffer containing 0.5% (v/v) Nonidet P-40, 150 mM NaCl, 50 mM Tris pH 7.4 and protease inhibitor cocktail (Sigma). Insoluble material was removed by centrifugation at 16,100× *g* for 20 min. Phosphatidylinositol-3-phosphate (PI3P/PtdIns3P) agarose beads (40 μl; Eschelon Biosciences) were incubated with cell lysates for 2 h at 4°C, followed by washing four times in buffer. Proteins were eluted from beads by heating at 98°C for 3 min in 2× sample loading buffer and then subjected to SDS-PAGE. Proteins binding to beads were detected by Western blotting using mouse anti-c-myc antibody (Sigma, 1:1000) and HRP-conjugated goat anti-mouse (Santa Cruz, 1:2000). Immunoreactivity was visualized using West Pico Chemiluminescent Substrate (Pierce). Quantification of PI3P pulldown assay results for myc-CB3_SH3-_, myc-CB3_SH3-_^R356Q^, myc-CB3_SH3-_^R290H^ and myc-CB3_SH3-_^R356N/R357N^ was performed in triplicate and differences in PI3P binding were assessed using an unpaired, two-tailed Student’s *t*-test.

### *In vitro* Gephyrin Clustering Assays

These were performed essentially as previously described (Long et al., 2016). HEK293 cells were co-transfected with the pRK5myc-hCB3_SH3-_^R356Q^ construct at a 1:1 ratio with pEGFP-gephyrin using electroporation (Gene Pulser II, Bio-Rad). Cells were fixed after 24 h for 2 min in 4% (w/v) PFA in PBS. Immunostaining to detect collybistin was performed using a mouse anti-c-myc antibody (1:200, Sigma) and detected using an AlexaFluor 546 goat anti-mouse secondary antibody (1:600; Invitrogen). Counterstaining for cell nuclei was performed with DAPI (1:500; Life Technologies). Confocal microscopy was performed using a Zeiss LSM 710 META. All images were taken with a ×63 objective.

### Neuronal Cell Culture, Transfections and Immunofluorescence

The sheep anti-GAD (lot 1440-4) antibody was a gift from Dr. Irwin J. Kopin (NINDS, Bethesda, MD, USA). This antibody, raised against purified rat GAD, recognizes a 65-kDa protein in rat brain immunoblots. The antibody precipitated GAD from rat brain and detected purified GAD in crossed immunoelectrophoresis (Oertel et al., 1981). The Rb antibody to gephyrin (catalog # 261003) was from Synaptic Systems (Gottingen, Germany). The mouse mAb to cMyc was from Millipore (Temecula, CA; clone 4A6, catalog no.05–724). Fluorophore-labeled species-specific anti-IgG cross-adsorbed secondary antibodies were made in donkey. The fluorescein isothiocyanate (FITC), or aminomethylcoumarin were from Jackson ImmunoResearch Laboratories, West Grove, PA and the AlexaFluor 594 was from Invitrogen. Hippocampal (HP) neuronal cultures were prepared according to Goslin et al. (1998) as described elsewhere (Christie et al., 2002a,b; Christie and De Blas, 2003). Briefly, dissociated neurons from embryonic day 18 (E18) rat hippocampi were plated (10,000–20,000 cells per 18 mm diameter coverslip) and maintained in rat glial cell conditioned medium. HP neurons (10 DIV) cultures were transfected with 1 μg of plasmid using the CalPhos Mammalian Transfection Kit (BD Biosciences, San Jose, CA, USA), according to the instructions provided by the manufacturer. Cultures were fixed 72 h later, permeabilized and subjected to immunofluorescence as described elsewhere (Christie et al., 2002a,b; Li et al., 2010, 2012; Chiou et al., 2011; Fekete et al., 2015). Fluorescence images of cultured hippocampal neurons were collected using a Nikon Plan Apo 60×/1.40 objective on a Nikon Eclipse T300 microscope with a Photometrics CoolSNAP HQ2 CCD camera driven by IPLab 4.0 (Scanalytics, Rockville, MD) acquisition software. Images were processed with Photoshop 7.0 (Adobe, San Jose, CA), adjusting brightness and contrast, as described elsewhere (Christie et al., 2002b; Li et al., 2005).

### Molecular Dynamics Simulations

Molecular dynamics (MD) simulations were performed using the GROMACS engine, version 2016.1 in conjunction with the GROMOS54A7 force field (Schmid et al., 2011; Abraham et al., 2015). The coordinates and parameters for PI3P were developed using the Automated Topology Builder (ATB) and Repository (Malde et al., 2011; Koziara et al., 2014) and are available for download from the ATB (molecule ID: 294885). The coordinates for the open conformation, wild-type collybistin (CB1_SH3-_) were taken from the protein data bank (PDB ID: 4MT7). The p.R356Q substitution was introduced computationally to produce a structural model of the collybistin mutant p.R356Q (collybistin^R356Q^). The PI3P head group was docked to both wild-type and mutant collybistin using Autodock vina (Trott and Olson, 2010) in the vicinity of R356, to provide a range of putative interaction conformations. The lowest energy docked conformation was used as a template to rebuild the intact PI3P molecule prior to MD simulations. Two separate systems were used to initiate MD simulation: PI3P complexed with wild-type collybistin and PI3P complexed with collybistin^R356Q^. Each system was explicitly solvated with SPC water molecules and Na^+^ counter-ions were added to ensure the overall charge neutrality of the system. A detailed description of the system set-up is provided as Supplementary Information. After energy minimization and equilibration, each system was simulated in triplicate for 200 ns. Following MD simulation, the triplicate simulations from the PI3P/collybistin system were combined to give a 600 ns trajectory containing 1,200 frames. Cluster analysis was performed on the backbone structure of the collybistin protein using the clustering algorithm described by Daura et al. (1999). Protein conformations were grouped using a 2.5 Å distance cut-off. Here, two conformations were considered to fall within the same cluster if the backbone RMSD between the conformations was less than the specified cut-off of 2.5 Å. This procedure was repeated for the PI3P/collybistin^R356Q^ system. The central (median) conformation from the top two most populated conformational clusters for the PI3P/collybistin system and the PI3P/collybistin^R356Q^ system were taken as representative conformations of PI3P-bound collybistin and collybistin^R356Q^. Protein residues within 3 Å of PI3P were considered binding residues and were determined using the Visual Molecular Dynamics (VMD) software (Humphrey et al., 1996).

## Results

### Identification of a p.R356Q Mutation in *ARHGEF9*

**Figure 1:**
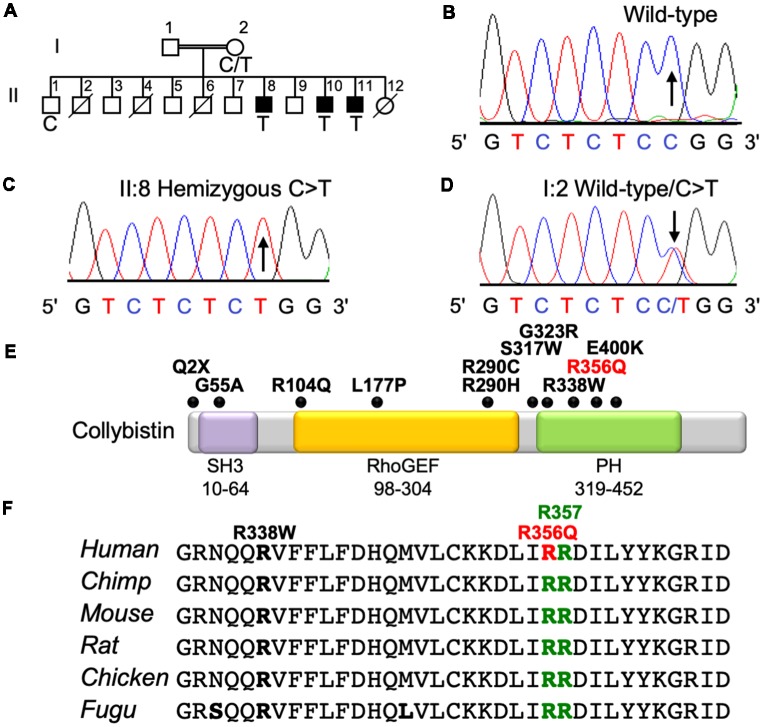
Identification of a p.R356Q mutation in *ARHGEF9* in family D228. **(A)** Pedigree of the D228 family. Open symbols represent normal individuals, filled squares represent affected males. Individuals who are deceased have a slash through the symbol. Individuals tested for the nucleotide substitution in each family are indicated with either a T (mutant allele) or a C (normal allele). **(B–D)** DNA sequence electropherograms for the chrX:62875607C>T, p.R356Q variant reported in this study, showing wild-type, hemizygous and heterozygous states.** (E)** Schematic of the human collybistin protein with a regulatory SH3 domain, a catalytic RhoGEF domain and a pleckstrin homology (PH) domain. The relative locations of known missense and nonsense variants in *ARHGEF9* are shown. **(F)** Sequence alignments of collybistin proteins from various species showing the high conservation of R356 in the PH domain. R356 is one of two predicted PI3P binding residues (R356 and R357, green highlighting).

The three affected males from this family presented with mild NS XLID (Figure 1A) without seizures, neurologic, ocular or other phenotypes. X-chromosome exome resequencing of individual II:8 followed by bioinformatics analysis and filtering against public datasets revealed four novel missense changes: *ARHGEF9*, chrX:62875607C>T, p.R356Q (Figures 1B–D), consensus score 5.13, predicted as probably damaging (PolyPhen-2; Adzhubei et al., 2013) and damaging (SIFT; Kumar et al., 2009) with a CADD score (Kircher et al., 2014) of 26; *CCDC22*, chrX: 49105321A>G, p.A492G with a conservation score of 5.56 and predicted as benign (Polyphen-2) and tolerated (SIFT) and CADD of 18; *FAM3A*, chrX:153736880T>C, p.E37G with a conservation score 2.67 and predicted as benign and tolerated and CADD of 15; *GLUD2*, chrX:120181572T>C, p.S12P with a low conservation score (0.06) and predicted as benign and damaging with a CADD score of 11. This strongly suggested that one of the novel missense variants identified in the established XLID genes *ARHGEF9* (Harvey et al., 2004; Marco et al., 2008; Kalscheuer et al., 2009; Lesca et al., 2011; Shimojima et al., 2011; Lemke et al., 2012; de Ligt et al., 2012; Long et al., 2016; Alber et al., 2017; Klein et al., 2017; Wang et al., 2018) and *CCDC22* (Voineagu et al., 2012; Kolanczyk et al., 2015) or a combination of both could be responsible for XLID in this family. Subsequent segregation analysis using Sanger DNA sequencing indicated that both variants were present in the three affected males (Figures 1A–D), and in heterozygous form in their mother, whereas the unaffected brother did not have these variants. Thus, both variants co-segregated with the XLID phenotype in all individuals tested. We subsequently performed functional assays on the *ARHGEF9* missense mutation, which had the highest CADD score.

### XLID Mutation p.R356Q Disrupts PH Domain—PI3P Interactions and Collybistin-Mediated Gephyrin Clustering in Cellular Assays

**Figure 2:**
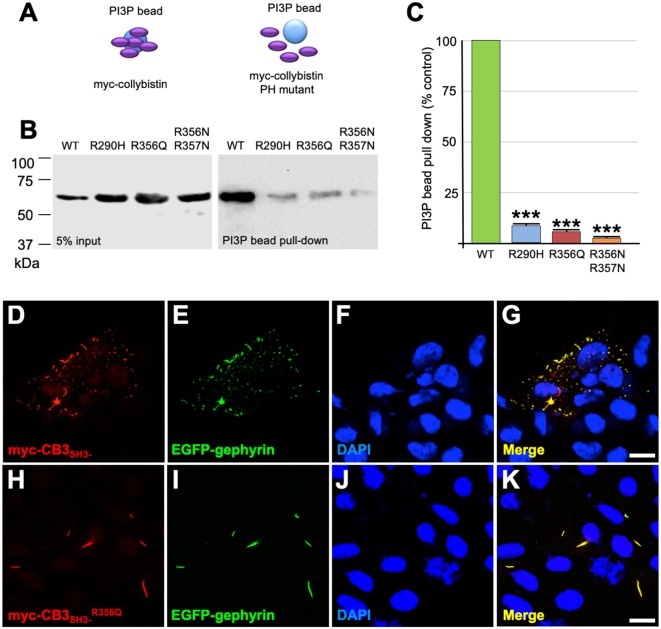
R356Q reduces collybistin PI3P binding and gephyrin cluster formation in cellular assays. **(A,B)** Human embryonic kidney 293 (HEK293) cells were transfected with plasmids encoding myc-CB3_SH3-_ (wild-type, WT), myc-CB3_SH3-_^R356Q^ (R356Q), myc-CB3_SH3-_^R290H^ (R290H) or myc-CB3_SH3-_^R356Q^ (R356N/R357N) and cell lysates were incubated with PI3P-conjugated agarose beads. After washing, bound material was subjected to SDS-PAGE and analyzed by immunoblotting with an anti-myc antibody (Sigma). No significant differences were observed in total level of myc-CB3_SH3-_^R356Q^, myc-CB3_SH3-_^R290H^ or myc-CB3_SH3-_^R356N/R357N^ expression in comparison with myc-CB3_SH3-_ (left panel, 5% input protein). However, myc-CB3_SH3-_^R356Q^ myc-CB3_SH3-_^R290H^ and myc-CB3_SH3-_^R356N/R357N^ binding to PI3P was significantly reduced (right panel, PI3P bead pull-down). **(C)** Quantification of PI3P pull-down assays normalized to wild-type myc-CB3_SH3-_: myc-CB3_SH3-_^R290H^: 8.73 ± 0.79, 1.37; myc-CB3_SH3-_^R356Q^: 7.62 ± 0.97, 1.68; myc-hCB3_SH3-_^R356N/R357N^: 3.41 ± 0.85, 1.47; pull-down fraction ± SEM, SD; *n* = 3, *** *p* < 0.0001, unpaired student’s *t*-test. **(D–K)** HEK293 cells were co-transfected with EGFP-gephyrin and either wild-type myc-CB3_SH3-_ or mutant myc-CB3_SH3-_^R356Q^, immunostained using anti-myc and AlexaFluor 546 antibodies and co-stained with a nuclear marker (DAPI). Note that while wild-type myc-CB3_SH3-_ (red) co-localizes with gephyrin (green) in submembrane microaggregates **(D–G)**, mutant myc-CB3_SH3-_^R356Q^ (red) co-localizes with EGFP-gephyrin (green) in elongated cytoplasmic aggregates **(H–K)**, consistent with a lack of CB-mediated gephyrin clustering activity for the p.R356Q mutant. Scale bar = 10 μm.

Collybistin has a multi-domain structure consisting of a regulatory SH3 domain, a catalytic RhoGEF domain and a PH domain (Figure 1E). Substitution p.R356Q is the first reported *ARHGEF9* missense mutation affecting a highly-conserved phosphoinositide-binding residue in the PH domain (Figure 1F). In order to determine whether the p.R356Q mutation affected collybistin binding to PI3P, we performed pulldown assays (Figures 2A,B) using PI3P immobilized on agarose beads incubated with lysates of HEK293 cells transfected with either tagged wild-type collybistin (myc-CB3_SH3-_), the new mutant myc-CB3_SH3-_^R356Q^, a known human XLID mutant known to be deficient in PI3P binding (myc-CB3_SH3-_^R290H^; Papadopoulos et al., 2015) or an artificial phosphoinositide binding site mutant myc-CB3_SH3-_^R356N/R357N^ (Reddy-Alla et al., 2010). A significant reduction of PI3P binding was observed for myc-CB3_SH3-_^R356Q^ (7.62 ± 0.97), myc-CB3_SH3-_^R290H^ (8.73 ± 0.79) and myc-hCB3_SH3-_^R356N/R357N^ (3.41 ± 0.85) compared to wild-type collybistin (Figures 2B,C; pull-down fraction ± SEM, *n* = 3, *p* < 0.0001, unpaired student’s *t*-test). Given the key role of collybistin in gephyrin clustering at inhibitory synapses, we also investigated whether the p.R356Q substitution influenced collybistin-mediated translocation of EGFP-gephyrin to submembrane microaggregates in a cellular clustering assay (Harvey et al., 2004; Kalscheuer et al., 2009; Long et al., 2016). Collybistin variants containing the regulatory SH3 domain (e.g., myc-CB3_SH3+_) typically co-localize with EGFP-gephyrin in large intracellular aggregates (Kins et al., 2000; Harvey et al., 2004; Kalscheuer et al., 2009; Long et al., 2016) and require neuroligin 2/4, GABA_A_R α2 or the GTPase TC10 for activation (Poulopoulos et al., 2009; Saiepour et al., 2010; Mayer et al., 2013). By contrast, variants lacking the N-terminal SH3 domain (e.g., myc-CB3_SH3-_) result in the formation of submembrane EGFP-gephyrin clusters (Kins et al., 2000; Harvey et al., 2004; Kalscheuer et al., 2009; Long et al., 2016; Figures 2D–G). On co-expression with EGFP-gephyrin, myc-CB3_SH3-_^R356Q^ was not capable of forming submembrane microaggregates, but co-localized with EGFP-gephyrin in elongated cytoplasmic aggregates (Figures 2H–K). This distribution is similar to that previously observed for collybistin variants lacking the PH domain (Harvey et al., 2004) or containing missense mutations that disrupt PI3P binding, such as p.R290H, p.R338W and p.R356N/R357N (Reddy-Alla et al., 2010; Papadopoulos et al., 2015; Long et al., 2016). These results demonstrate that the p.R356Q substitution disrupts both collybistin PI3P binding and collybistin-mediated accumulation of EGFP-gephyrin in submembrane microclusters.

### Collybistin Phosphoinositide Binding Mutants p.R356Q and p.R356N/R357N Alter Gephyrin Cluster Number and Size in Cultured Hippocampal Neurones

**Figure 3:**
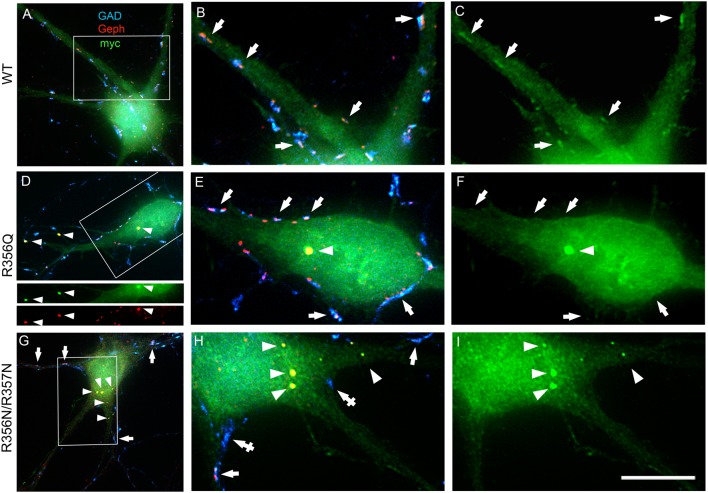
Collybistin phosphoinositide binding mutants R356Q and R356N/R357N alter gephyrin cluster number and size in cultured hippocampal neurones. Triple-label immunofluorescence of transfected hippocampal neurons with mouse anti-myc (green), rabbit anti-gephyrin (red) and sheep anti-GAD (blue). **(A–C)** Transfection with myc-CB3_SH3-_ (wild-type, WT). **(D–F)** Transfection with myc-CB3_SH3-_^R356Q^ (R356Q). **(G–I)** Transfection with myc-CB3_SH3-_^R356N/R357N^ (R356N/R357N). The two panels on the right side of each row correspond to the boxed areas in **(A,D,G)**, respectively. Arrows point to GABAergic synapses. Arrowheads point to aggregates of gephyrin that form in transfections with collybistin R356Q or R356N/R357N. Note that there is colocalization of mutant collybistin and gephyrin in these aggregates. Wild-type collybistin does not induce the formation of aggregates but accumulates at GABAergic synapses and colocalizes with gephyrin (arrows, panel **C**). The R356Q and the R356N/R357N mutants do not accumulate at synapses (arrows, panels **F,I**). The R356N/R357N mutant (panels **H,I**) shows a clear decrease in the overall number of gephyrin clusters, including synaptic gephyrin clusters. In neurons overexpressing the R356N/R357N mutant, GAD positive puncta often make contacts without apposed gephyrin clusters (crossed arrows). Scale bar = 26 μm for **(A,D,G)** and 10 μm for **(B,C,E,F,H,I)**.

To assess the impact of the p.R356Q mutation on collybistin-mediated neuronal gephyrin clustering, we overexpressed either wild-type myc-CB3_SH3-_, myc-CB3_SH3-_^R356Q^ or the artificial PI3P binding mutant myc-CB3_SH3-_^R356N/R357N^ in primary cultures of rat hippocampal neurons. Previous studies in this neuronal expression system have shown that wild-type recombinant collybistin isoforms (e.g., CB2_SH3+_, CB2_SH3-_, CB3_SH3+_, and CB3_SH3-_) target to and concentrate at GABAergic postsynapses (Chiou et al., 2011). Notably, isoforms lacking the SH3 domain (e.g., CB2_SH3-_ and CB3_SH3-_) induce the formation of synaptic gephyrin superclusters (Chiou et al., 2011; Fekete et al., 2017) that are accompanied by a significant increase in the amplitude of miniature inhibitory postsynaptic currents (mIPSCs). In this study, we utilized triple-label immunofluorescence with mouse anti-myc, rabbit anti-gephyrin and sheep anti-GAD to reveal exogenous wild-type and mutant collybistin, native gephyrin and GAD, respectively (Figures 3A–C). As expected, overexpressed wild-type collybistin (myc-CB3_SH3-_; Figures 3A–C) accumulated at GABAergic synapses, colocalizing with gephyrin and GAD65 (Figures 3B,C, arrows). By contrast, overexpression of myc-CB3_SH3-_^R356Q^ and myc-CB3_SH3-_^R356N/R357N^ (Figures 3D–I) resulted in the formation of non-synaptic aggregates (Figures 3D–I, arrowheads) characterized by a circular shape and bright fluorescence. Mutant myc-CB3_SH3-_^R356Q^ and myc-CB3_SH3-_^R356N/R357N^ in these aggregates co-localized with gephyrin (see, e.g., Figure 3D, lower panels) but did not associate with GAD-positive terminals (Figures 3D–I). Hence, myc-CB3_SH3-_^R356Q^ and myc-CB3_SH3-_^R356N/R357N^ do not accumulate at inhibitory synapses (arrows in Figures 3F,I). We also noted that in neurons overexpressing the myc-CB3_SH3-_^R356N/R357N^ mutant, GAD-positive puncta often make contacts without apposed gephyrin clusters (Figure 3H, crossed arrows)—an effect that was not seen for myc-CB3_SH3-_ or myc-CB3_SH3-_^R356Q^.

**Figure 4:**
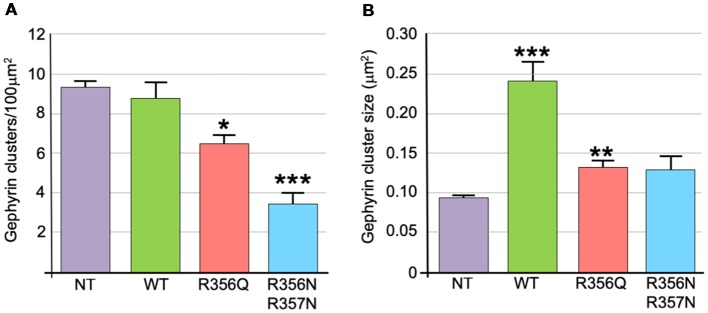
Quantification of gephyrin cluster number and cluster size for wild-type collybistin and mutants R356Q and R356N/R357N in cultured hippocampal neurones. **(A)** To quantify the effect of these mutants, we measured gephyrin cluster density/100 μm^2^ in non-transfected neurones (NT = 9.33 ± 0.33, 1.04) and neurones transfected with wild-type collybistin (WT = 8.73 ± 0.88, 2.77) and collybistin phosphoinositide binding mutants (R356Q = 6.42 ± 0.52, 1.65; R356N/R357N = 3.37 ± 0.63, 1.99; mean ± SEM, SD). The gephyrin cluster density was decreased for R356Q but not as much as for R356N/R357N, *p < 0.05; ***p < 0.001 for each mutant respectively compared with either NT or WT, *n* = 10 neurons each. The total number of clusters counted for density were 283, 273, 189 and 99 for NT, WT, R356Q and R356N/R357N, respectively. For quantification of cluster density, two independent transfections were made for each construct, and all constructs were used in each experiment. A total of 60 dendritic fields (50 μm^2^ each) per construct from 10 randomly selected neurons (10 neurons/construct, 3 dendrites/neuron, 2 dendritic fields/dendrite) were analyzed. **(B)** Gephyrin cluster size was NT = 0.095 ± 0.003, 0.011; WT = 0.240 ± 0.024, 0.076; R356Q = 0.132 ± 0.008, 0.026; R356N/R357N = 0.129 ± 0.016, 0.051. Values are mean ± SEM, SD / μm^2^, *n* = 10 neurons each. For quantification of cluster size, two independent transfections were made for each construct, and all constructs were used in each experiment. ***p < 0.001 for WT with each of the other plasmids; **p < 0.01 for R356Q compared with NT indicates a slight increase in cluster size in this mutant. The total number of clusters counted for size were 680, 584, 492 and 180 for NT, WT, R356Q and R356N/R357N, respectively. No significant difference was observed for R356N/R357N compared with NT or R356Q. The data were analyzed by one-way analysis of variance with a Tukey-Kramer multiple comparisons test.

We also assessed the effect of wild-type and mutant collybistin isoforms on synaptic gephyrin cluster density (Figure 4A) and size (Figure 4B), excluding the large non-synaptic gephyrin/mutant aggregates (for myc-CB3_SH3-_^R356Q^ and myc-CB3_SH3-_^R356N/R357N^) from this analysis. No significant differences in cluster density were observed comparing non-transfected neurones with those expressing wild-type myc-CB3_SH3-_ (non-transfected = 9.33 ± 0.33 vs. myc-CB3_SH3-_ = 8.73 ± 0.88; mean clusters/100 μm^2^ ± SEM). However, gephyrin cluster density was significantly decreased for myc-CB3_SH3-_^R356Q^ (6.42 ± 0.52 clusters/100 μm^2^) with a further decrease for myc-CB3_SH3-_^R356N/R357N^ (3.37 ± 0.63 clusters/100 μm^2^). As expected from previous studies (Chiou et al., 2011), synaptic gephyrin cluster size was significantly increased on overexpression of myc-CB3_SH3-_ (non-transfected = 0.095 ± 0.003 vs. wild-type myc-CB3_SH3-_ = 0.240 ± 0.024; mean clusters μm^2^ ± SEM). However, only a slight increase in cluster size was observed for myc-CB3_SH3-_^R356Q^ (0.132 ± 0.008) with no significant difference observed for myc-CB3_SH3-_^R356N/R357N^ (0.129 ± 0.016) compared non-transfected neurones.

### Molecular Dynamics Simulations Reveal That Wild-Type Collybistin and Collybistin^R356Q^ Display Distinct Conformations That May Underlie Observed Differences in PI3P Binding

**Figure 5:**
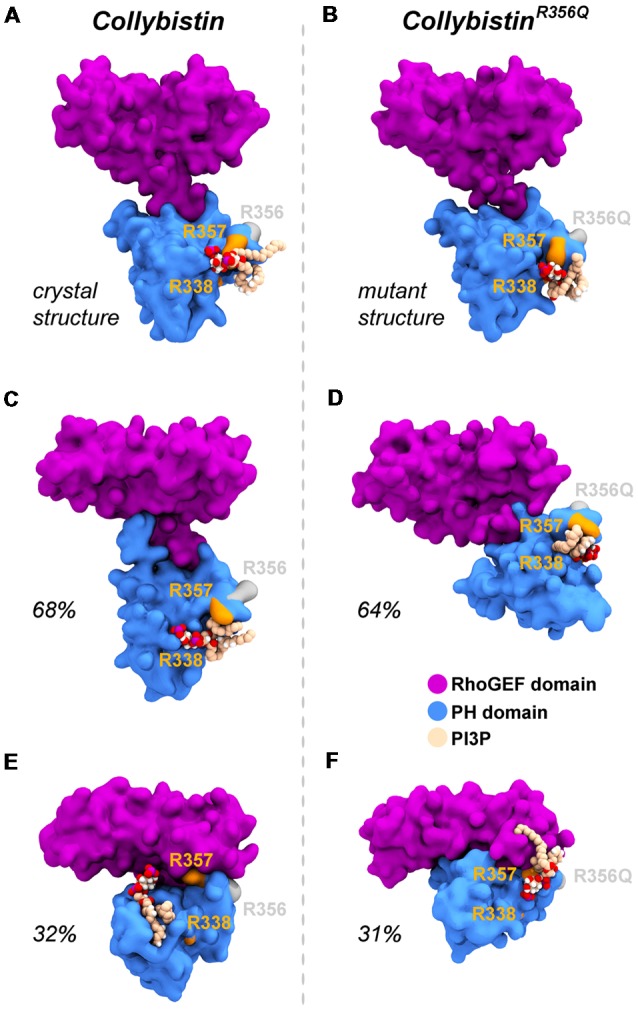
Wild-type collybistin and collybistin^R356Q^ adopt two distinct conformations in solution with unique PI3P binding sites. PI3P (shown in space-fill representation, colored by atom where carbon = tan, oxygen = red, nitrogen = blue, hydrogen = white and phosphorous = purple) is docked to wild-type collybistin **(A)** and collybistin^R356Q^ **(B)** structures (collybistin is shown as a purple and blue surface). Representative collybistin conformations and PI3P binding poses from the most frequently adopted conformations during molecular dynamics simulations are shown in panels **(C–F)**. A 2.5 Å cut-off distance was used to perform the cluster analysis and the proportion of the simulation that each conformation represents is labeled. Key arginine residues R338, R357 and R356 are colored orange (R338 and R357) and gray (R356).

**Figure 6:**
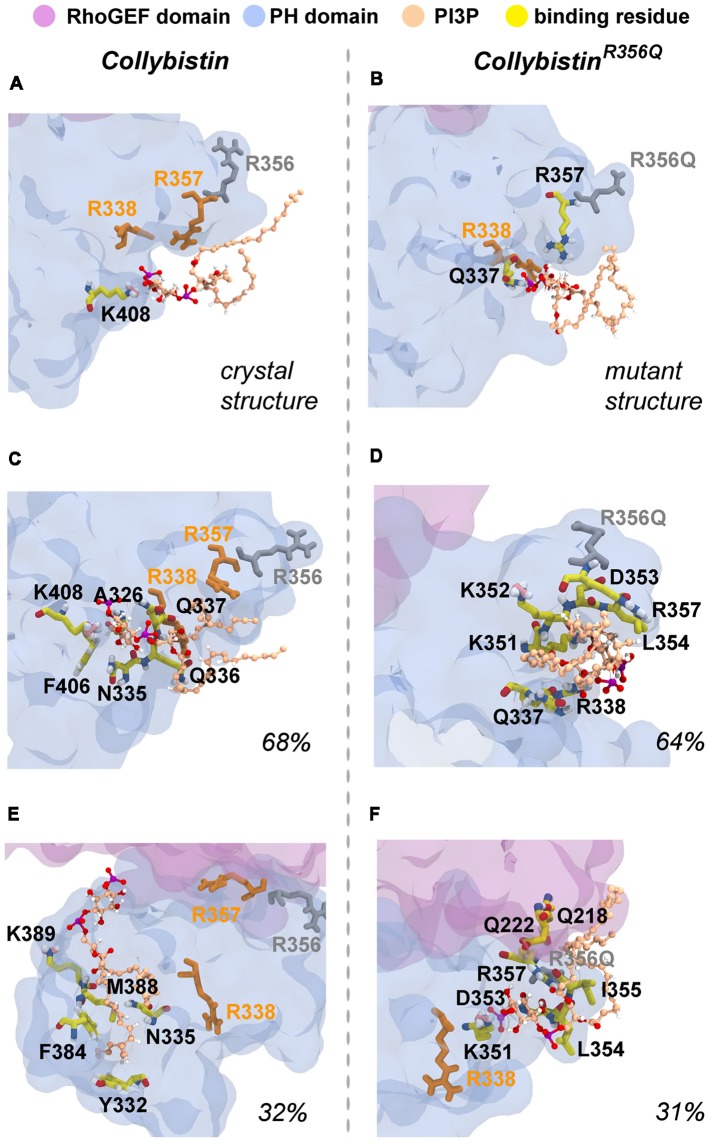
Predicted PI3P-binding residues in wild-type collybistin and collybistin^R356Q^. Predicted PI3P binding residues in the protein conformation obtained from PI3P docking to **(A)** the wild-type collybistin crystal structure (PDB ID: 4MT7) and **(B)** the computational model of the collybistin^R365Q^ mutation. PI3P binding residues from MD simulations of the most populated conformational cluster of **(C)** wild-type collybistin and **(D)** collybistin^R365Q^. PI3P binding residues from MD simulations of the second most populated conformational cluster of **(E)** wild-type collybistin and** (F)** collybistin^R365Q^. The PH and RhoGEF domains are shown in transparent gray-blue and mauve, respectively. R357 and R338 are shown in gold licorice. R556 and R356Q are shown as gray licorice. The PI3P lipid is shown as a ball-and-stick model, colored by atom (carbon = tan, oxygen = red, nitrogen = blue, hydrogen = white and phosphorous = purple). The PI3P-binding residues, taken as those within 3 Å of the PI3P molecule, are shown in stick representation, colored by atom (carbon = yellow, oxygen = red, nitrogen = blue, hydrogen = white, pink = resonance structure). Key arginine residues that impact phosphoinositide binding, including R338 (Long et al., 2016), R356 and R357 (Reddy-Alla et al., 2010) are shown as sticks and are colored orange (R338 and R357) and gray (R356). All residues shown are labeled, with binding residues labeled with black lettering.

MD simulations revealed that both wild-type and mutant collybistin^R356Q^ adopted a range of conformations that could be grouped into two distinct conformational clusters. These differed from the reported crystallographic conformation of wild-type collybistin and the mutant collybistin^R356Q^ model with docked PI3P (Figures 5A,B), which were used as starting structures to initiate the simulations. Cluster analysis of wild-type collybistin showed that it adopted a more elongated conformation for approximately 68% (814 out of 1200 structures) and an “open clam” conformation for approximately 32% (380 out of 1200 structures) of the combined trajectory. The central conformations of each are shown in Figures 5C,E, respectively. Cluster analysis of collybistin^R356Q^ also revealed two distinct conformations during the combined trajectories, that differed from the conformations adopted by wild-type collybistin. Collybistin^R356Q^ predominantly displayed a “side saddle” conformation for 64% (Figure 5D, 769 out of 1,200 structures) and a “shut clam” conformation for 31% (Figure 5F, 373 out of 1,200 structures) of the combined simulation trajectory. During MD simulations, both the position and orientation of the PI3P molecule changed significantly from its initial docked location in both wild-type collybistin and collybistin^R356Q^ simulations (Figures 5A,B, respectively), binding to a unique site in the PH domain in each conformational cluster, as shown in Figure 5. Table 1 and Figure 6 show that there was little overlap in the residues that bind PI3P in each of the predominant conformational clusters. There was more overlap in the PI3P-binding residues in the starting conformation of collybistin^R356Q^ and its two predominant conformational clusters than in the representative conformations than for wild-type collybistin. Intriguingly, in the second most populated conformational cluster of wild-type collybistin, PI3P moves far from the initial docked location to bind in the deep binding cleft of the “open clam” conformation shown in Figures 5E, 6E.

**Table 1:** The PI3P-binding residues in molecular dynamics simulations of wild-type collybistin and collybistin^R356Q^.

	Putative PI3P-binding residues	
	Wild-type collybistin	collybistin^R356Q^
Crystal structure	K408	Q337
		R357
Most prevalent conformation (~60%)	A326	Q337
	N335	R338
	Q336	K351
	Q337	K352
	F406	D353
	K408	L354
		R357
Second most prevalent conformation (~30%)	Y332	Q218
	N335	Q222
	F384	K351
	M388	D353
	K389	L354
		I355
		R357

## Discussion

This study reports the identification and functional characterization of a novel PH domain mutation (p.R356Q) in the RhoGEF collybistin that is likely to represent the cause of XLID in this family. Using X-exome sequencing, bioinformatics analysis and inheritance testing, we found a novel SNV (chrX:62875607C>T, p.R356Q) in *ARHGEF9* that segregated with the disease phenotype. This substitution of a highly conserved functionally important residue in the PH domain had a consensus score of 5.13, and was predicted as probably damaging and damaging by PolyPhen-2 and SIFT, with a CADD score of 26. Although we did not exclude a further potential variant in *CCDC22*, a gene that has previously been linked to ID, this substitution was predicted as benign and tolerated by Polyphen-2 and SIFT and had a lower CADD score of 18. It is also noteworthy that missense mutations in *CCDC22* are associated with Ritscher-Schinzel syndrome-2 (Kolanczyk et al., 2015) an X-linked recessive syndromic form of ID associated with posterior fossa defects, cardiac malformations, and minor abnormalities of the face and distal extremities. These additional phenotypic features have not been observed in the family under study to date.

Using multiple biochemical and cellular functional assays, we were able to establish the likely pathomechanism for collybistin p.R356Q—a reduction in PI3P binding in pulldown assays, leading to a loss of gephyrin clustering activity in an *in vitro* EGFP-gephyrin clustering assay (Harvey et al., 2004). It is noteworthy that collybistin constructs tested in this study represent CB3_SH3-_, i.e., lack the N-terminal SH3 domain, because isoforms containing the SH3 domain do not show EGFP-clustering activity in HEK293 cells without the addition of additional regulatory proteins such as neuroligin 2 or the GABA_A_R α2 subunit (Harvey et al., 2004; Poulopoulos et al., 2009; Saiepour et al., 2010). In the absence of these interactors, collybistin isoforms harboring the N-terminal SH3 domain (e.g., CB3_SH3+_) adopts a closed and autoinhibited conformation that prevents the PH domain from binding to phosphoinositides (Ludolphs et al., 2016). Hence, we used CB3_SH3-_ rather than CB3_SH3+_ in our PI3P pull-down and cellular clustering assays. In cultured hippocampal neurones, all tested collybistin isoforms (CB2_SH3+_, CB2_SH3-_, CB3_SH3+_ and CB3_SH3-_) target to and concentrate at GABAergic postsynapses (Chiou et al., 2011; Fekete et al., 2017) with the major difference being that transfection of de-regulated isoforms that lack the SH3 domain (e.g., CB3_SH3-_) results in large postsynaptic gephyrin and GABA_A_ receptor superclusters, while isoforms containing the SH3 domain induce the formation of supernumerary non-synaptic clusters (Chiou et al., 2011; Fekete et al., 2017). In this system, overexpression of the myc-CB3_SH3-_^R356Q^ mutant resulted in a decrease in the overall number of synaptic gephyrin clusters compared to controls. Notably, p.R356Q also abrogated the ability of myc-CB3_SH3-_ to form gephyrin superclusters (Chiou et al., 2011; Fekete et al., 2017). Taken together, our analysis strongly suggests that the collybistin p.R356Q variant is responsible for the mild intellectual disability observed in this family—due to disruption of collybistin PI3P binding, leading to a decrease in synaptic gephyrin and GABA_A_ receptor clustering.

A number of mutations in *ARHGEF9* have been identified in patients encompassing missense and nonsense mutations, deletions and complex rearrangements (Long et al., 2016; Alber et al., 2017). As noted by recent reviews aimed at correlating genotypes and patient phenotypes (Alber et al., 2017; Wang et al., 2018), the associated patient phenotypes vary quite substantially. Individuals with mutations in *ARHGEF9* present in early childhood, with delayed motor development sometimes in combination with seizures. Intellectual disability generally ranges from moderate to severe, although males with severe intellectual disability often have intractable epilepsy and facial dysmorphism, including enlarged, fleshy earlobes, a sunken appearance of the middle face in combination with a protruding of the jaw (Alber et al., 2017). Curiously, several patients with mutations affecting the PH domain (p.R338W, Long et al., 2016; p.E400K, de Ligt et al., 2012; p.R356Q, this study) appear to have mild or moderate XLID and do not develop seizures. A further PH domain mutation close to the RhoGEF-PH linker, p.G323R, also resulted in a low frequency of delayed onset febrile and afebrile seizures (Klein et al., 2017).

So why do PH domain mutations appear to have less impact? There are several possibilities as to why this might be the case. Certainly, mutations that affect the N-terminal SH3 domain have the potential to be extremely deleterious, either unlocking collybistin into a conformationally “open” state (Soykan et al., 2014; Ludolphs et al., 2016), or disrupting key synaptic interactors. For example, the collybistin mutation p.G55A associated with early infantile epileptic encephalopathy and severe psychomotor retardation has a clear dominant-negative effect, causing gephyrin aggregation in neurons and subsequent loss of synaptic gephyrin and GABA_A_ receptor clusters (Harvey et al., 2004). The p.G55A mutation also disrupts collybistin interactions with key synaptic molecules that interface with the SH3 domain, including neuroligin 2 (Poulopoulos et al., 2009) and GABA_A_ receptor α2 subunit (Saiepour et al., 2010). By contrast, mutations found in the RhoGEF domain could potentially affect GDP-GTP exchange on the small GTPase Cdc42 (Reddy-Alla et al., 2010), gephyrin binding (Xiang et al., 2006) or alter the strength of intramolecular interactions RhoGEF and PH domains, as in the case of the p.R290H mutation (Papadopoulos et al., 2015) that indirectly affected PI3P binding. To date, two of the reported PH domain mutations (p.R338W, Long et al., 2016; p.R356Q, this study) appear to operate by reducing the affinity of collybistin for phosphoinositides such as PI3P, although interactions with the small Rho-like GTPase TC10—which also binds to the collybistin PH domain (Mayer et al., 2013)—have not yet been assessed. Our MD simulations show that wild-type collybistin adopted at least two distinct conformations in solution (Figures 5C,D). These are distinct from the two conformations adopted by mutant collybistin^R356Q^ (Figures 5E,F). Furthermore, PI3P did not remain bound at the docking location during the simulations, but instead bound in unique locations on the PH domain that depended on the protein conformation (Figure 6). Our MD results suggest that the range of conformations adopted by wild-type collybistin are likely to be impacted by the p.R356Q variant, and hence affect PI3P binding. Indeed, there was little overlap in the PI3P-binding residues for wild-type collybistin, as shown in Table 1 and Figure 6. However, residue Q337 bound PI3P in both of the most prevalent conformations of collybistin and is likely to be an important PI3P-binding residue.

Although the p.R356Q and p.R338W substitutions completely inhibit collybistin-mediated gephyrin clustering in cellular models (Figure 2; Long et al., 2016), the effects observed for p.R356Q in primary neurones were more modest. Although overexpression of myc-CB3_SH3-_^R356Q^ resulted in the formation of non-synaptic collybistin-gephyrin aggregates, CB3_SH3-_^R356Q^ was not able to localize to synaptic sites and is therefore unlikely to have dominant-negative effects. Rather, CB3_SH3-_^R356Q^ modestly reduced synaptic gephyrin cluster density, without affecting cluster size (Figure 3). Under these circumstances, we would predict that missense mutations in the collybistin PH domain that disrupt PI3P binding will show milder disease course than dominant-negative collybistin mutations in the SH3 or RhoGEF domains that substantially reduce gephyrin cluster number and size, so disrupting associated inhibitory GABAergic transmission.

## Author Contributions

RH, VK, MO, ADB and VMK designed the experiments. T-TC, PL, AS-G, VK and KH performed the experiments. RH, VK, MO, SH, ADB and VMK analyzed the data. RH, VK, AS-G, MO, ADB and VMK wrote the article. All authors were involved in revising the article for important intellectual content, and gave final approval of the version to be published.

## Conflict of Interest Statement

The authors declare that the research was conducted in the absence of any commercial or financial relationships that could be construed as a potential conflict of interest.
