# Supplementary material for: Mutation p.R356Q in the Collybistin Phosphoinositide Binding Site Is Associated With Mild Intellectual Disability
Source: Front Mol Neurosci. 2019 Mar 12;12:60. doi: 10.3389/fnmol.2019.00060 (PMC6422930; doi:10.3389/fnmol.2019.00060)
Supplement: Supplementary file 1 [file Table_1.DOCX]

**Mutation p.R356Q in the collybistin phosphoinositide**

**binding site is associated with mild intellectual disability**

**Tzu-Ting Chiou^1^, Philip Long^2^, Alexandra Schumann-Gillett^3^,**

**Venkateswarlu Kanamarlapudi^4^, Stefan A. Haas^5^, Kirsten Harvey^2^,**

**Megan L. O’Mara^3^, Angel L. De Blas^1^, Vera M. Kalscheuer^6^, Robert J. Harvey^7,8^**

^1^Department of Physiology and Neurobiology, University of Connecticut, Storrs, Connecticut, 06269, USA; ^2^Department of Pharmacology, UCL School of Pharmacy, London WC1N 1AX, United Kingdom; ^3^Research School of Chemistry, The Australian National University, Canberra, ACT 2601, Australia; ^4^Institute of Life Science, School of Medicine, Swansea University, Singleton Park, Swansea SA2 8PP, United Kingdom; ^5^Max Planck Institute for Molecular Genetics, Department of Computational Molecular Biology, D-14195 Berlin, Germany;  ^6^Max Planck Institute for Molecular Genetics, Group Development and Disease, D-14195 Berlin, Germany; ^7^School of Health and Sport Sciences, University of the Sunshine Coast, Sippy Downs, QLD 4556, Australia; ^8^Sunshine Coast Health Institute, Birtinya, QLD 4575, Australia.

**Computational molecular geometries and topologies**

The crystal structure of open wild-type collybistin (CB1_SH3-_; PDB ID: 4MT7) was used for computational modelling. The R356Q variant was introduced into wild-type collybistin using PyMOL (DeLano, 2014). The structure and molecular topology of PI3P were generated using the Automated Topology Builder (ATB) (Malde et al., 2011; Koziara et al., 2014) (molecule identification number 294885). To ensure that the *cis* double bonds in the PI3P molecule remained in the *cis* conformation and did not rotate to the *trans* conformation during the simulations, the force constant associated with each *cis* double bond dihedral angle was altered from 5.86 kJ/mol/rad^2^ to 41.80 kJ/mol/rad^2^.

**PI3P docking and molecular dynamics simulations setup**

The head group of PI3P (PI3P that ­lacked the glycerol and bound acyl chains) was docked to wild-type collybistin and collybistin^R356Q^ using Autodock vina (Trott and Olson, 2010). The docking search space was defined as a box around residue R356 for both wild-type collybistin and collybistin^R356Q^. Docking was performed with 10 points in the x-direction, 16 points in the y-direction and 16 points in the z-direction. The entire PI3P molecule was superimposed on the lowest energy docking conformation. The N- and C-termini of the proteins were capped with neutral acetal and amine groups, respectively. All simulations were performed using the GROMACS package, version 2016.1 in conjunction with the GROMOS 54A7 force field (Schmid et al., 2011; Abraham et al., 2015). Each docked collybistin/PI3P complex was solvated with simple point charge water molecules in a rectangular box, and Na^+^ counter ions were added to neutralise the charge. Periodic boundary conditions were applied. The minimum distance between the collybistin/PI3P complex and its periodic image was 3 nm. The system was energy minimised using a steepest descent algorithm. It was then equilibrated during a series of five 1 ns simulations, where the positions of the backbone atoms of the protein were restrained using sequentially descending force constants of 1000 kJ mol^-1^ nm^-1^, 500 kJ mol^-1^ nm^-1^, 100 kJ mol^-1^ nm^-1^, 50 kJ mol^-1^ nm^-1^ and 10 kJ mol^-1^ nm^-1^.

**Molecular dynamics simulation details**

Both the collybistin/PI3P and collybistin^R356Q^/PI3P docked complexes were simulated in triplicate for 200 ns, unrestrained. A unique starting velocity was assigned to each replicate simulation. The LINCS algorithm (Hess et al., 1997) was used to constrain the covalent bond lengths, and the short- and long-range cut-off values were 0.8 nm and 1.4 nm respectively. To minimise the effect of truncating the electrostatics beyond the long-range cut-off, a reaction-field correction with a dielectric constant of ε_r_ = 78.5 was applied. The geometry of the water molecules was constrained using the SETTLE algorithm (Miyamoto and Kollman, 1992). The equations of motion were integrated every 2 fs and the simulations were performed in the NPT ensemble. The temperature was maintained close to 310 K by weakly coupling the system to an external temperature bath. A 0.1 ps temperature coupling relaxation time constant was used. Similar to the temperature, the pressure was maintained near 1 bar by weakly coupling the system to an external pressure bath. Isotropic pressure coupling was used, with a compressibility of 4.5 × 10^-5^ bar and a pressure coupling relaxation time constant of 1 ps. The coordinates of the atoms were saved every 500 ps. Molecular images were created using the Visual Molecular Dynamics (VMD) software (Humphrey et al., 1996).

**Cluster analysis**

For both wild-type collybistin and collybistin^R356Q^ systems, the three 200 ns simulations were combined into a single trajectory file. Every tenth frame was removed in both data sets, so that each of the two concatenated 600 ns trajectories had 1200 frames (note that each 200 ns trajectory had 400 frames after removing the tenth frame). Root-mean-square cluster analysis was performed on the backbone of the protein for the combined trajectories, using a 2.5 Å cut-off distance. The coordinates of the middle structure from the two most populated conformations were used as a representative structure of that conformation, as shown in Figure 5.

**PI3P-binding residues**

Collybistin residues that were within 3Å of PI3P were determined using VMD (Humphrey et al., 1996). This analysis was performed for wild-type collybistin and collybistin^R356Q^ PI3P-docked crystal structures and the representative conformations from the most populated conformations. PDB files for PI3P docked to collybistin and collybistin^R356Q^ are available on request from Megan O'Mara (E-mail: megan.o'mara@anu.edu.au).

**Primary data for PI3P pull-down assay Western blots (Fig. 2)**

| **Replicate** | **Wild-type (%)** | **R356Q** | **R290H** | **R356N/R357N** |
| --- | --- | --- | --- | --- |
| 1 | 100 | 5.82 | 10.16 | 2.45 |
| 2 | 100 | 9.15 | 8.59 | 5.10 |
| 3 | 100 | 7.90 | 7.43 | 2.67 |
| Mean | - | 7.62 | 8.73 | 3.41 |
| SEM | - | 0.97 | 0.79 | 0.85 |
| SD | - | 1.68 | 1.37 | 1.47 |

**Primary data for neuronal gephyrin density/100 µm^2^ and cluster size (Fig. 3, Fig. 4)**

| **Density/100 µm^2^** | **Non-transfected** | **Wild-type CB** | **R356Q** | **R356N/R357N** |
| --- | --- | --- | --- | --- |
| Mean | 9.33 | 8.73 | 6.42 | 3.37 |
| SEM | 0.33 | 0.88 | 0.52 | 0.63 |
| SD | 1.04 | 2.77 | 1.65 | 1.99 |
| Clusters counted | 283 | 273 | 189 | 99 |

| **Cluster size** | **Non-transfected** | **Wild-type CB** | **R356Q** | **R356N/R357N** |
| --- | --- | --- | --- | --- |
| Mean | 0.095 | 0.240 | 0.132 | 0.129 |
| SEM | 0.003 | 0.024 | 0.008 | 0.016 |
| SD | 0.011 | 0.076 | 0.026 | 0.051 |
| Clusters counted | 680 | 584 | 492 | 180 |

**References**

Abraham, M.J., Murtola, T., Schulz, R., Páll, S., Smith, J.C., Hess, B., et al. (2015) Gromacs: high performance molecular simulations through multi-level parallelism from laptops to supercomputers. SoftwareX 1-2, 19-25. doi:10.1016/j.softx.2015.06.001

DeLano, W.L. (2014) The PyMOL Molecular Graphics System, Version 1.8. Schrödinger LLC. http://www.pymol.org. doi:10.1038/hr.2014.17

Hess, B., Bekker, H., Berendsen, H.J.C., Fraaije, J.G.E.M. (1997) LINCS: A Linear Constraint Solver for molecular simulations. *J. Comput. Chem.* 18, 1463-1472. doi:10.1002/(SICI)1096-987X(199709)18:12<1463::AID-JCC4>3.0.CO;2-H

Humphrey, W., Dalke, A., Schulten, K. (1996) VMD: visual molecular dynamics. *J. Mol. Graph.* 14, 33-38. doi:10.1016/0263-7855(96)00018-5

Koziara, K.B., Stroet, M., Malde, A.K., Mark, A.E. (2014) Testing and validation of the Automated Topology Builder (ATB) version 2.0: prediction of hydration free enthalpies. *J. Comput. Aided. Mol. Des.* 28, 221-233. doi:10.1007/s10822-014-9713-7

Malde, A.K., Zuo, L., Breeze, M., Stroet, M., Poger, D., Nair, P.C., et al. (2011) An Automated force field Topology Builder (ATB) and repository: version 1.0. *J. Chem. Theory Comput.* 7, 4026-4037. doi:10.1021/ct200196m

Miyamoto, S., Kollman, P.A. (1992) Settle: an analytical version of the SHAKE and RATTLE algorithm for rigid water models. *J. Comput. Chem.* 13, 952-962. doi:10.1002/jcc.540130805

Schmid, N., Eichenberger, A.P., Choutko, A., Riniker, S., Winger, M., Mark, A.E., et al. (2011) Definition and testing of the GROMOS force-field versions 54A7 and 54B7. *Eur. Biophys. J.* 40, 843-856. doi:10.1007/s00249-011-0700-9

Trott, O., Olson, A.J. (2010) AutoDock Vina. *J. Comput. Chem.* 31, 445-461. doi:10.1002/jcc.21334
